# Chamaejasmine Inactivates Akt To Trigger Apoptosis in Human HEp-2 Larynx Carcinoma Cells

**DOI:** 10.3390/molecules16108152

**Published:** 2011-09-27

**Authors:** Yu Wang, Yan Zhao, Ying Liu, Linli Tian, Dejun Jin

**Affiliations:** 1 Department of Otorhinolaryngology–Head and Neck Surgery, The Second Affiliated Hospital of Harbin Medical University, Harbin 150001, China; E-Mails: xberry@hljucm.net (Y.W.); appleliuying@yahoo.com.cn (Y.L.); tianlinli78@hotmail.com (L.T.); 2 Department of Otorhinolaryngology, The First Affiliated Hospital of Harbin Medical University, Harbin 150001, China; E-Mail: zhaoyanhaerbin@yahoo.com.cn

**Keywords:** chamaejasmine, HEp-2, Akt, *in vivo*, apoptosis

## Abstract

In the present study, we investigated the mechanisms of chamaejasmine action on human HEp-2 larynx carcinoma cells, which possess constitutively active Akt. Results indicated that chamaejasmine showed more notable anticancer activity than apigenin against HEp-2, PC-3, NCI-H1975, HT-29 and SKOV-3. Moreover, chamaejasmine presented most significantly inhibition towards HEp-2, with IC_50_ values of 1.92 µM. Treatment of HEp-2 cells with chamaejasmine (1–4 μM) resulted in significant dose-dependent decrease in Akt phosphorylation at Serine473. Chamaejasmine-mediated dephosphorylation of Akt resulted in inhibition of its kinase activity, which was confirmed by reduced phosphorylation of proapoptotic proteins BAD and glycogen synthase kinase-3, essential downstream targets of Akt. Inactivation of Akt seems to be associated with downregulation of insulin-like growth factor receptor 1 protein level and inhibition of its autophosphorylation upon chamaejasmine treatment. Exposure to chamaejasmine significantly induced caspase-9 and caspase-3 activity. *In vivo*, chamaejasmine intake through gavage resulted in inactivation of Akt and induction of apoptosis in HEp-2 tumors. These results suggest that Akt inactivation and dephosphorylation of BAD is a critical event, at least in part, in chamaejasmine-induced HEp-2 cells apoptosis.

## 1. Introduction

Laryngeal carcinoma, the third most common cancer worldwide, causes substantial morbidity and mortality every year [1]. During the last two decades, many approaches had been applied to treat this cancer, but the survival rate has not considerably improved [2,3]. The traditional treatment for primary laryngeal carcinoma is surgical resection, particularly total laryngectomy for patients with advanced disease [4]. Nowadays, with more emphasis on the quality of life of cancer patients, novel strategies show a shift from pure survival to maximum preservation of laryngeal function. Accordingly, a large number of chemotherapeutic agents are currently in use. However, these drugs are highly toxic and have a low survival profile [5]. Thus, an understanding of the molecular mechanisms of more effective and less harmful therapies are needed in the treatment of laryngeal carcinoma.

Akt, also called protein kinase B (PKB), is the human homologue of the viral oncogene v-Akt. Akt is an evolutionary conserved serine/threonine kinase [6,7]. Conserved from primitive metazoans to humans, the Akt subfamily comprises three closely related, highly conserved mammalian isoforms: Akt1, Akt2 and Akt3, which are also called PKBα, PKBβ and PKBγ, respectively. Akt1 is involved in cellular survival pathways, by inhibiting apoptotic processes. Akt2 is an important signaling molecule in the insulin signaling pathway, where it is required to induce glucose transport. The role of Akt3 is less clear, though it appears to be predominantly expressed in brain [8,9,10]. These three serine/threonine kinases are products of distinct genes and share a conserved structure that includes three functional domains: an N-terminal pleckstrin homology domain, a central kinase domain and a C-terminal regulatory domain containing the hydrophobic motif phosphorylation site [7].

The phosphatidylinositol-3-kinase (PI3K) signaling pathway is crucial to many aspects of cell growth and survival. It is targeted by genomic aberrations including mutation, amplification and rearrangement more frequently than any other pathway in human cancer [11]. Genetic screens in model organisms have identified AKT as the primary downstream mediator of the effects of PI3K. The PI3K/AKT and related pathways are important in internalizing the effects of external growth factors and of membrane tyrosine kinases. Activation of membrane kinases including epidermal growth factor receptor (EGFR) by external growth factors initiates receptor dimerization and subsequent events to activate these intracellular pathways [12,13]. PI3K catalyzes the formation of lipid second messenger phosphatidylinositol-3,4,5-triphosphate (PIP3) at the cell membrane by phosphorylation of phosphatidylinositol-4,5-bisphosphate in a reaction that can be reversed by the tumor suppressor phosphatase and tensin homologue deleted on chromosome 10 (PTEN), a negative regulator of the PI3K–Akt-signaling pathway [14]. The N-terminal pleckstrin homology domain in the N-terminal region of Akt interacts with PIP3 at the plasma membrane resulting in the recruitment of Akt to the plasma membrane. Recruitment to the membrane results in a conformational change that exposes two crucial amino acids that are phosphorylated and necessary for activation: one in the kinase domains (threonine 308 in AKT1) is phosphorylated by constitutively active phosphoinositide-dependent kinase 1 (PDK1), stabilizing the activation loop, whereas phosphorylation of the other in the hydrophobic C-terminal domain (serine 473 in AKT1) by PDK2 is necessary for full activation [15].

Once activated, Akt transduces signals from growth factors and oncogenes to downstream targets that control tumor-associated crucial cellular processes, including cell growth, cell cycle progression, survival, migration, tissue invasion and angiogenesis [16,17]. Frequent deregulation of the PI3K–Akt pathway in cancer has prompted significant interest in blocking this pathway to prevent and/or treat cancer [18,19,20], as specific inhibition of the activation of Akt by small molecules may be a valid approach to prevent and/or treat human malignancies.

**Figure 1:**
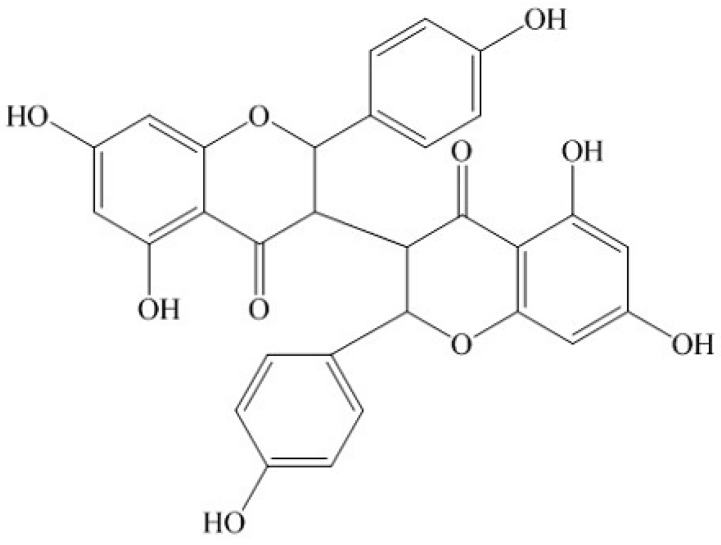
Chemical structure of chamaejasmine.

*Stellera chamaejasme* L. belongs to the Thymealaeaceae, widely distributed in northwest and southwest China. The roots of *Stellera chamaejasme* L. can be used as a pesticide on bugs, flies and maggots, and also to control pests on crops, and pastures [21,22]. It has been found that the methanol extracts of the root of *Stellera chamaejasme* L. showed significant antitumor activities [23]. Chamaejasmine (Figure 1) is a natural biflavanone with notable antitumor and pesticidal activity [24,25]. It was one of the major biflavanones obtained from *Stellera chamaejasme* L. To our best knowledge, the anticancer activity of chamaejasmine against HEp-2 has not been elucidated yet and this study thus represents the investigation of the effects of chamaejasmine against HEp-2 cells.

In the present study, human HEp-2 larynx carcinoma cells as well as laryngeal cancer xenografts in athymic nude mice were studied to investigate the effects of chamaejasmine on Akt inactivation. Our findings provide experimental evidence indicating that chamaejasmine-induced decreased cell survival and apoptosis in HEp-2 cells are mediated by inactivation of Akt, leading to BAD dephosphorylation and activation of caspase-9 and caspase-3.

## 2. Results and Discussion

### 2.1. Cytotoxicity Assays

The cytotoxicity of chamaejasmine was evaluated on five human cancer cell lines (HEp-2, PC-3, NCI-H1975, HT-29, SKOV-3) and one normal cell lines (HEK293) using MTT assays. Apigenin was used as positive control. The results were listed in Table 1. Chamaejasmine exhibited stronger inhibition against all five cancer cell lines than apigenin. Among all of them, chamaejasmine showed more notable cytotoxicity against HEp-2 than PC-3, NCI-H1975, HT-29 and SKOV-3, with IC_50_ values of 1.92, 3.61, 14.30, 10.67 and 8.04 µM, respectively. Though the cytotoxicity of apigenin was 5-fold lower than chamaejasmine against HEK293, its anti-HEp-2 activity was 12-fold lower than chamaejasmine. Additionally, DMSO (6.4 × 10^−3^% − 0.05 × 10^−3^%) which used as vehicle didn’t show any toxicity against all cell lines.

Fang *et al.* studied the anticancer activity of chamaejasmine against nine human cancer cell lines (MCF-7, A549, SGC-7901, HCT-8, HO-4980, Hela, HepG2, PC-3 and LNCap) and two normal cell lines (Vero and MDCK) by MTT assays. Their results showed that chamaejasmine exhibited strong cytotoxicity against all nine cancer cell lines, with IC_50_ values ranging from 2.28 to 14.36 µM [25]. Combined with our results, we can conclude that chamaejasmine presents broad spectrum antitumor activity. There have been some reports on the anti-tumor activity of biflavones against various human cancer cell lines, suggesting that they may be promising candidates for novel anticancer agents [26]. Amentoflavone, obtained from *Selaginella tamariscina*, showed certain antitumor activity against Hela and MCF-7 cell lines, with IC_50_ values of 76.83 µM and 67.71 µM, respectively, that have led it to be considered as a potential anticancer agent [26]. The IC_50_ values of chamaejasmine were much lower, which suggests a more promising potential use of chamaejasmine in cancer therapy than that of amentoflavone.

### 2.2. Chamaejasmine Inhibits Akt Ser473 Phosphorylation and Downstream Targets

**Figure 2:**
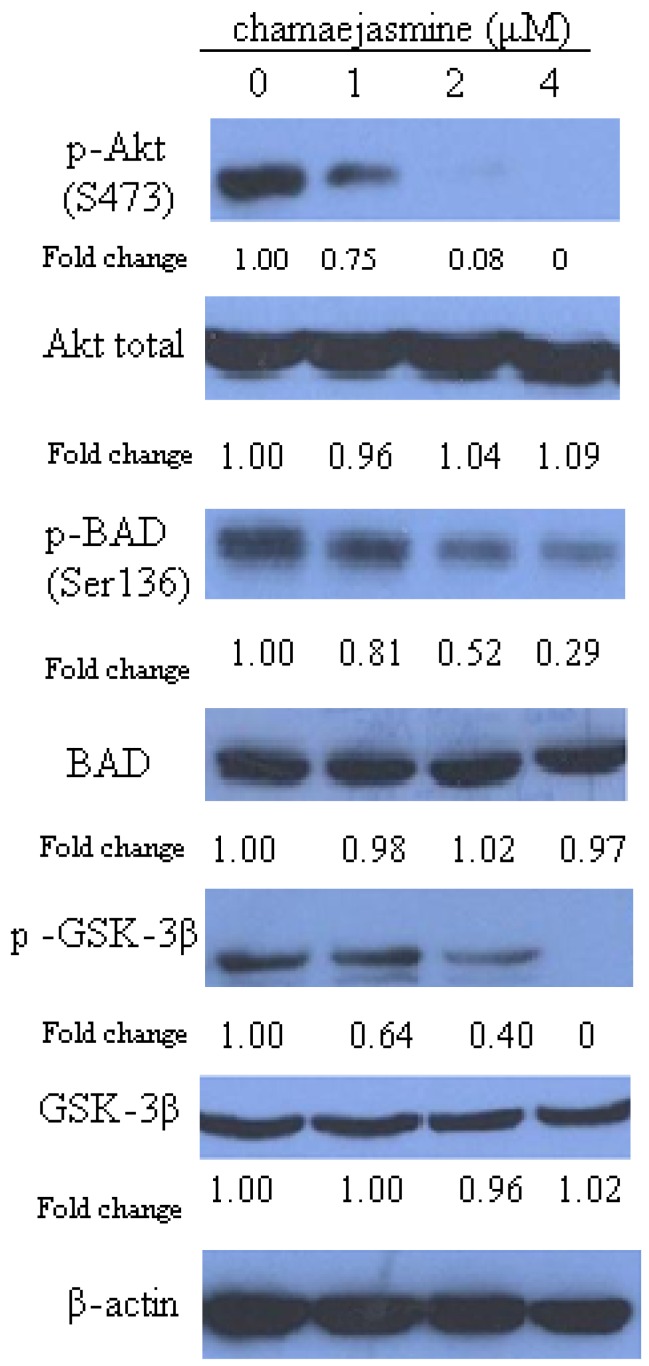
Immunoblotting for phosphor-Ser(473)-Akt, Akt, phospho-BAD, BAD, p-GSK-3β and GSK-3β using lysates from HEp-2 cells treated with chamejasmine (0, 1 µM, 2 µM and 4 µM) for 48 h. The blots were stripped and reprobed with anti-β-actin antibody to normalize the protein loading. Bands were quantitated by densitometric analysis. Fold change represents the protein level of the chamejasmine-treated cells relative to the control cells treated with vehicle and the resulting protein levels were then normalized to the β-actin protein.

As shown in Figure 2, treatment of HEp-2 cells with chamaejasmine decreased Ser473 phosphorylation of Akt significantly and in a concentration-dependent manner (25%, 92% and 100% upon treatment with 1, 2 and 4 µM chamaejasmine, respectively; *p* < 0.05). We further examined whether downstream targets of Akt are also affected by the decreased phosphorylation of Akt. Phosphorylation of glycogen synthase kinase-3 (GSK3)β, which is a well-recognized direct target of Akt [27], was obviously decreased upon chamaejasmine treatment (*p* < 0.05). This finding further confirmed the inhibition of Akt kinase activity by chamaejasmine treatment. Consistently, phosphorylation of BAD was decreased significantly as a result of decreased phosphorylation and kinase activity of Akt (by 19% at 1 µM, 48% at 2 µM and 71% at 4 µM chamaejasmine treatment; *p* < 0.05). However, the level of Akt total, BAD and GSK-3β was not affected even after 4 µM chamaejasmine treatment (*p* > 0.05).

The PI3K–Akt pathway has been found to be activated in various human cancers [28]. Activation of cells by growth factors or cytokines leads to recruitment of PI3K to the plasma membrane, where it catalyzes the conversion of phosphatidylinositol-4,5-bisphosphate in the D3 position to generate PIP3. The accumulation of PIP3 creates a docking site for Akt at the plasma membrane with conformational change in the Akt that exposes two crucial amino acids, threonine 308 in the kinase domain and serine 473 in the hydrophobic motif domain of Akt1 [15,28]. Phosphorylation at Ser473 was found to activate Akt to phosphorylate its targets through its kinase activity to promote survival and inhibit apoptosis [13,14,15]. Based on our results, chamaejasmine treatment causes an obvious decrease in activating phosphorylations of Akt at Ser473 in HEp-2 cells coupled with inhibition of GSK-3β and BAD phosphorylation, demonstrating that chamaejasmine inhibits Akt Ser473 phosphorylation and downstream targets of the Akt-signaling pathway.

### 2.3. Chamaejasmine Causes Downregulation of IGF-IR Protein and Its Phosphorylation

**Figure 3:**
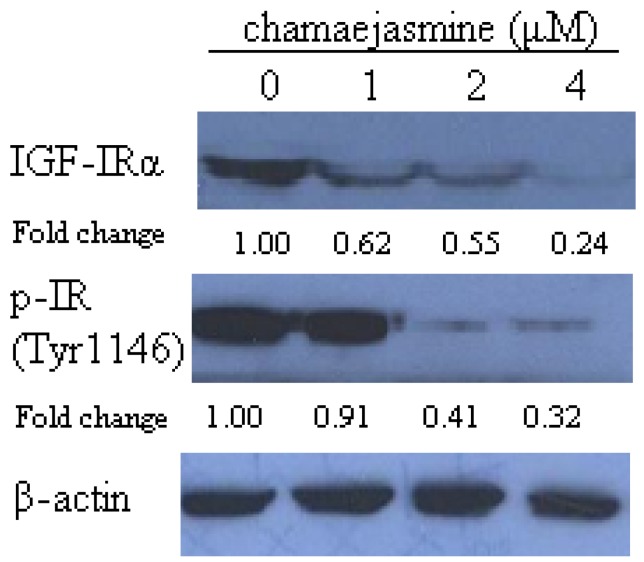
Immunoblotting for IGF-I Receptor α and phospho-IGF-IF (Tyr1131)/Insulin Receptor (Tyr1146) using lysates from HEp-2 cells treated with 0, 1, 2 and 4 μM chamaejasmine. The blots were stripped and reprobed with anti-β-actin antibody to normalize protein loading. Change was calculated as described in Figure 2.

The effect of chamejasmine on insulin like growth factor receptor (IGF-IR) expression and its autophoshorylation was further studied to explore the effects on phosphorylation of Akt of chamejasmine treatment. As shown in Figure 3, chamejasmine reduced the IGF-IR protein expression level by 38%, 45% and 76% upon 1, 2 and 4 µM chamejasmine treatment, respectively. The IGF-IR phosphorylation was downregulated by 9% at 1 µM, 59% at 2 µM and 68% at 4 µM chamejasmine treatment.

It has been found that activation of AKT is associated with the up-regulation of IGF-IR expression [29]. Numerous studies have demonstrated that overexpression and excessive activation of IGF-IR are associated with malignant transformations, increased tumor aggressiveness, and protection from apoptosis [30,31,32]. Since chamejasmine could downregulate the expression of IGF-IR and IGF-IR phosphorylation, it may play a critical role in tumor treatment.

### 2.4. Chamaejasmine Induces Activation of Caspase-9 and Caspase-3

**Figure 4:**
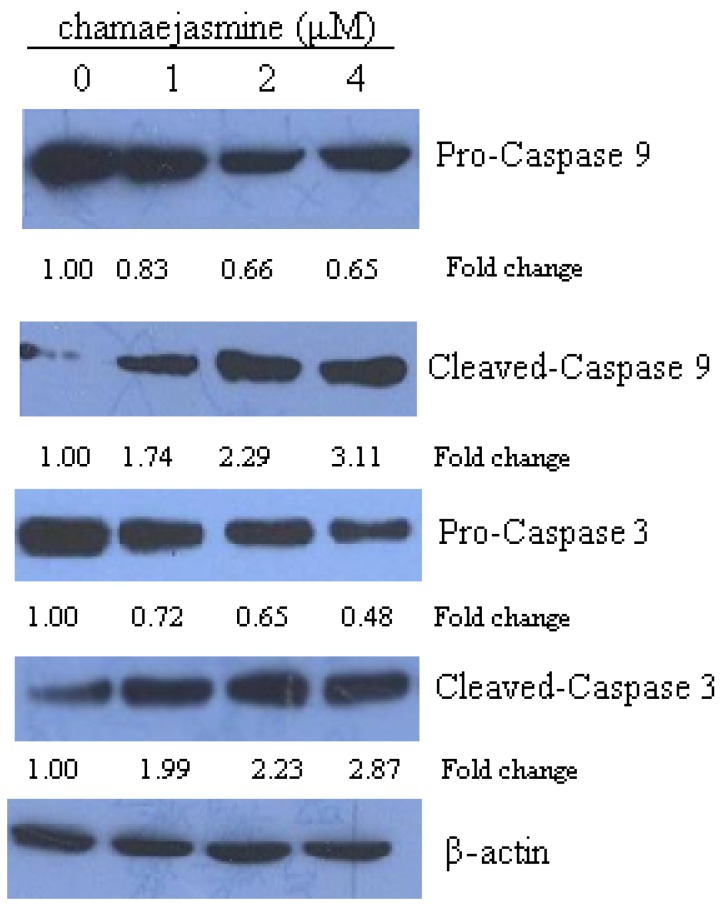
HEp-2 cells were exposed to 0, 1, 2 and 4 μM chamaejasmine for 48 h, cell lysates were prepared and immunoblotted for procaspase-9, cleaved caspase-9, procaspase-3 and cleaved caspase-9. Change was calculated as described in Figure 2.

In the present study activation of caspases was analyzed to ascertain whether chamaejasmine increased cell death through apoptosis. Treatment of HEp-2 cells with increasing concentrations of chamaejasmine elevated the protein expression of cleaved caspase-9 and caspase-3 (Figure 4).

The phosphorylation of caspase family member caspase-9 (Mch-6/ICE-LAP6) at Ser196 by Akt has been reported as another mechanism of Akt-mediated cell survival [33]. Caspase-9 may activate caspase-3, which is a well-known downstream adaptor caspase. Caspase-3 initiated the apoptotic cascade that leads to the cleavage and inactivation of key cellular proteins such as PARP [34,35].

### 2.5. Chamaejasmine Intake Inhibits Growth of HEp-2 Xenografts in Athymic Nude Mice

**Figure 5:**
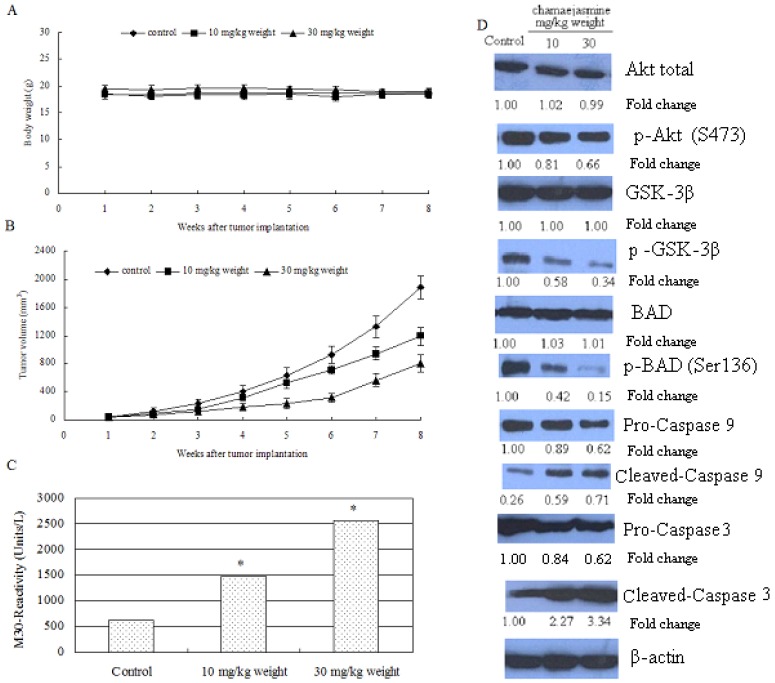
Chamaejasmine intake by gavage inhibits HEp-2 tumor growth in athymic nude mice via inactivation of Akt/PKB-signaling pathways. Chamaejasmine was provided to the animals two weeks after cell inoculation. Chamaejasmine was provided orally with 0.5% methyl cellulose and 0.025% Tween 20 as vehicle to these animals on a daily basis. Group I, control, received 0.2 mL vehicle only, Group II received 10 mg/kg weight chamaejasmine and Group III received 30 mg/kg weight chamaejasmine daily for eight weeks. (**A**) (**B**) Tumor volume (mm^3^) in control and treated groups. (**C**) Quantitative measurement of apoptosis as demonstrated by M30 reactivity for HEp-2 tumors after chamaejasmine intake at the indicated doses. * p < 0.05. (**D**) Immunoblots for Akt, phospho-Ser(473)-Akt, GSK-3β, p-GSK-3β, BAD, p-BAD-Ser136, pro and cleaved caspase-9, pro and cleaved caspase-3 in tumor lysates after chamaejasmine intake at the indicated doses. The blots were stripped and reprobed with anti-β-actin antibody to ensure equal protein loading.

Chamaejasmine has been proven to be effective against many cancer lines, inactivating Akt Ser473 phosphorylation and downstream targets (GSK and BAD, *etc*.) in HEp-2 cells. Therefore, a xenograft mouse model was further used to determine whether these events occur *in vivo*. Chamaejasmine was provided at 10 and 30 mg/kg weight daily through gavage, beginning two weeks after cell inoculation and continuing for eight weeks. Results showed that intake of chamaejasmine inhibited the growth of tumor xenograft at both doses of chamaejasmine. As shown in Figure 5A, chamaejasmine intake by these mice did not seem to induce any toxic effects, as judged by monitoring body weight. Tumor volume was inhibited by 35.3% and 64.7% (P < 0.05) respectively, at the termination of the experiment (Figure 5B). 

The induction of apoptosis in tumor xenografts induced by chamaejasmine intake was studied next. As shown in Figure 5C, daily oral intake of chamaejasmine at doses of 10 and 30 mg/kg weight resulted in a marked induction of apoptosis in HEp-2 tumor xenografts, as shown by M-30 reactivity measured by enzyme-linked immunosorbent assay. Compared with vehicle-treated control, 2.39- and 4.12-fold increases (P < 0.05) in the induction of apoptosis were observed in HEp-2 tumors after chamaejasmine treatment. Furthermore, consistent with the findings in cell culture, chamaejasmine administration to nude mice resulted in a dose-dependent decrease in the expression of p-Akt Ser473, p-GSK3β, p-BADSer136, with concomitant increases in cleaved caspase-9 and caspase-3, compared with mice receiving vehicle treatment. These results suggest that Akt inactivation and dephosphorylation of BAD causes induction of apoptosis in HEp-2 tumor xenografts (Figure 5D). To sum up, chamaejasmine resulted in inactivation of Akt through its reduced phosphorylation at Ser473 and its downstream targets p-BAD at Ser136 and p-GSK3β, increase in the levels of cleaved caspase-9 and caspase-3 favoring apoptosis *in vitro* and *in vivo*.

## 3. Experimental

### 3.1. Growth of Cell and Chemicals 

The human HEp-2 larynx carcinoma cells, human prostatic carcinoma cell line PC-3, non small cell lung cancer NCI-H1975, the human HT-29 colon adenocarcinoma cell line, ovarian carcinoma continuous cell line SKOV-3 and Human embryonic kidney cell line HEK293 were purchased from Harbin Medical University, (Harbin, China). HEp-2 were maintained in a 1:1 mix of Ham’s F12 and Dulbecco’s modified Eagle’s medium (DMEM) with 10% fetal bovine serum, 100 U of penicillin, 100 mg/mL streptomycin. PC-3, H1975, HT-29 and SKOV-3 were maintained in RPMI 1640 medium supplemented with 10% fetal bovine serum and 100 U/mL penicillin and 100 µg/mL streptomycin. The cells were kept at 37 °C in a humidified atmosphere containing 5% CO_2_. Chamaejasmine (molecular weight: 542.49) and apigenin (purity ≥ 99%, molecular weight: 270.25) were obtained from Sigma Chemical Co. (St. Louis, MO, USA). A 10 mM stock solution of chamaejasmine and apigenin was prepared in 1% dimethyl sulfoxide (DMSO) and stored at −80 °C. [3-(4,5)-dimethylthiazoly1)-3,5-diphenytetrazolium bromide (MTT) was also obtained from Sigma–Aldrich Inc. Deionized water was used in all experiments.

### 3.2. Cytotoxicity Assay

Inhibition of cell proliferation of chamaejasmine was measured by the MTT assay [36]. Briefly, cells were plated in 96-well culture plates (1 × 10^5^ cells/well) separately. After 24 h incubation, cells were treated with chamaejasmine or apigenin (0.5, 1, 2, 4, 8, 16, 32 and 64 µM, four wells per concentration) for 48 h, MTT solution (5 mg/mL) was then added to each well. Accordingly, DMSO (6.4 × 10^−3^% − 0.05 × 10^−3^%) which used as vehicle was also tested. After 4 h incubation, the formazan precipitate was dissolved in dimethyl sulfoxide (100 µL), and then the absorbance was measured in an ELISA reader (Thermo Molecular Devices Co., Union City, CA, USA) at 570 nm. The cell viability ratio was calculated by the following formula: Inhibitory ratio (%) = [(OD_control_ − OD_treated_)/(OD_control_)] × 100%. Cytotoxicity was expressed as the concentration of chamaejasmine inhibiting cell growth by 50% (IC_50_ value).

### 3.3. Western Blot Assays

To evaluate the expression levels of various intracellular proteins related to apoptosis, HEp-2 cells were treated with chamaejasmine (0, 1, 2 and 4 µM) for 48 h, respectively. For isolation of total protein fractions, cells were collected, washed twice with ice-cold Phosphate Buffered Saline (PBS), and lysed using cell lysis buffer [20 mM Tris pH 7.5, 150 mM NaCl, 1% Triton X-100, 2.5 mM sodium pyrophosphate, 1 mM EDTA, 1% Na_2_CO_3_, 0.5 µg/mL leupeptin, 1 mM phenylmethane-sulfonyl fluoride (PMSF)]. The lysates were collected by scraping from the plates and then centrifuged at 10,000 rpm at 4 °C for 5 min. Total protein samples (20 µg) were loaded on a 12% of SDS polyacrylamide gel for electrophoresis, and transferred onto polyvinylidene fluoride (PVDF) transfer membranes (Millipore, Billerica, MA, USA) at 0.8 mA/cm^2^ for 2 h. Membranes were blocked at room temperature for 2 h with blocking solution (1% BSA in PBS plus 0.05% Tween-20). Membranes were incubated overnight at 4 °C with primary antibodies (Calbiochem, San Diego, CA, USA, Cat#CP01), p-Akt Ser^473^ (Cat#9275), BAD (Cat#9292), p-BAD Ser^136^ (Cat#9295) caspase-3 (Cat#9662) caspase-9 (Cat#9502), GSK-3β (Cat#9332), p-GSK-3α/β Ser^21/9^ (Cat#9331), IGF-IRα (Cat#3022), p-IGF-IF Tyr^1131^ (Insulin Receptor, Tyr^1146^, Cat#3021), cleaved PARPAsp^214^ (Cat#9544) from Cell Signaling Technology (Beverly, MA) and Akt1/2 (Cat#SC-8312) and p-Akt Ser ^473^ (Cat#SC-7985-R) from Santa Cruz Biotechnologies, (Santa Criz, CA, USA) (mouse polyclonal antibodies) at a 1:1,000 dilution in blocking solution. After washing thrice in Tris Buffer Solution Tween (TBST) for 5 min each time, membranes were incubated for 1 h at room temperature with an alkaline phosphatase peroxidase conjugated anti-mouse secondary antibody at a dilution of 1:500 in blocking solution. Detection was performed by the BCIP/NBT Alkaline Phosphatase Color Development Kit (Beyotime Institute of Biotechnology, Shanghai, China) according to the manufacturer’s instructions. Bands were recorded with a digital camera (Nikon, Tokyo, Japan).

### 3.4. Tumor Xenograft Studies

The HEp-2 cell line was used to induce xenografts in 6-week-old athymic (nu/nu) nude female mice. Exponentially growing cells were harvested, washed with PBS, and resuspended in DMEM, and 1 × 10^6^ viable cells were transplanted subcutaneously into the right flank of mice. The 30 animals used were equally divided into three groups. The first group received only 0.2 mL of vehicle material by gavage daily and served as a control group. The second and third groups of animals received 10 and 30 mg/kg weight/day doses of chamaejasmine in vehicle, respectively, for eight weeks, starting two weeks after cell inoculation. Animals were monitored daily, and their body weights were recorded weekly throughout the studies. Once the tumors started growing, their sizes were measured thrice weekly in two dimensions with calipers. At the termination of the experiment, a portion of the tumors from control and treated animals was used for preparation of tumor lysate used in further experiments.

### 3.5. M-30 Apoptosis by Enzyme-Linked Immunosorbent Assay

Apoptosis was assessed by M30-ApoptosenseTM ELISA kit (Alexis Biochemicals, San Diego, CA, USA) according to the manufacturer’s protocol and color developed was read at 450 nm against the blank and values were plotted against standards provided and expressed as units per liter.

### 3.6. Statistical Analysis

The data were expressed as mean ± S.D. The statistical significance of the differences between treatment groups was determined by one-way ANOVA using the STATISTICA 6.0 software (StatSoft, Tulsa, OK, USA). A *p*-value of <0.05 was considered as significant. 

## 4. Conclusions

In summary, the present study showed that chamaejasmine most probably exerts its potential cancer preventive/therapeutic effects directly through the PI3K–Akt-signaling pathway. The demonstration of downregulation of constitutive Akt kinase activity by chamaejasmine provides a rationale to explore its role as a preventive and perhaps as a chemotherapeutic agent in the management of laryngeal carcinoma.

## Figures and Tables

**Table 1:** Inhibition concentrations 50% (IC_50_) values for chamaejasmine towards HEp-2, PC-3, H1975, HT-29, SKOV-3 and HEK293 cells determined by MTT assay.

Cell lines	IC_50_ (µM)	
	Chamaejasmine	Apigenin
HEp-2	1.92 *	24.17
PC-3	3.61 *	29.85
NCI-H1975	14.30 *	50.23
HT-29	10.67 *	40.96
SKOV-3	8.04 *	33.52
HEK293	3.16 *	16.79

* Statistically different from positive control (apigenin) (*p* < 0.05).
